# Thyroid Fine-Needle Aspiration and Smearing Techniques

**DOI:** 10.1089/ve.2018.0119

**Published:** 2018-06-07

**Authors:** Mitsuyoshi Hirokawa, Ayana Suzuki, Akira Miyauchi

**Affiliations:** Department of Diagnostic Pathology and Cytology, Kuma Hospital, Kobe, Japan.; Department of Clinical Laboratory, Kuma Hospital, Kobe, Japan.; Department of Surgery, Kuma Hospital, Kobe, Japan.

**Keywords:** fine-needle aspiration, smearing technique, thyroid, press and release method

## Abstract

***Introduction:*** Thyroid fine-needle aspiration cytology is the most reliable preoperative diagnostic tool, but cases of failed or unsatisfactory diagnostic can occur. Therefore, we aim to improve aspiration and smearing techniques. We handle approximately 8000 thyroid fine-needle aspiration cytology cases annually. Here, we present the aspiration and smearing techniques resulting from our accumulated experience.

***Materials and Methods:*** Patients undergo aspiration cytology while seated on a barber chair, and are asked to gaze upwards to extend their anterior neck.^1^ Instead of relying on suction force, the samples are mainly obtained by cutting the tissue with needle movements. A strong negative pressure and a long aspiration time frequently produce bloody samples. Hence, we recommend negative pressure <0.3 mL and aspiration time up to 3 seconds. The obtained samples are placed on a glass slide and smeared using a second slide glass through a press and release method. When the samples are bloody, we tilt the glass slide, remove excess material, and wipe up the bloody components flowing from the slide. Liquid-based cytology is especially recommended for bloody or fluid samples. Biochemical measurement of thyroglobulin and calcitonin using fine-needle washout fluids is useful for diagnosing metastatic differentiated thyroid carcinoma and medullary thyroid carcinoma.^2,3^ When lymphoma is suspected, flow cytometry using aspirated samples is recommended.^4^

***Results:*** By applying the mentioned techniques and recommendations, we observed an increased accuracy in diagnosis and improved quality of our examinations.

***Conclusions:*** Fine-needle aspiration requires aspirating from the areas suitable for the diagnosis, obtaining adequate materials, and performing optimal smearing and fixation to retrieve highly accurate diagnoses. We hope our methods are helpful in improving your fine-needle aspiration cytology techniques, and result in more accurate cytological diagnoses. Thank you for taking interest in our methods.

**Acknowledgment:** We would like to thank Dr. Louise Davies, Associate Professor of Surgery—Otolaryngology—Head and Neck Surgery, Geisel School of Medicine at Dartmouth, Hanover, NH, for her valuable comments on the creation of this video.

We have no connection to any companies or products mentioned in this content. No competing financial interests exist.

Runtime of video: 7 mins 15 secs

The Japanese version of this video was presented at the 29th Annual Congress of the Japan Association of Endocrine Surgeons, held in May 18, 2017, in Kobe, Japan.

## Graphical abstract

**Figure:**
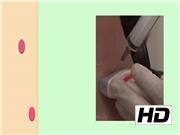

**Figure:**
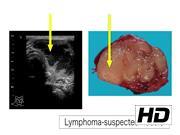

**Figure:**
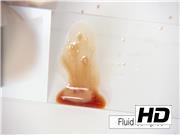

## Video

## Files

**MP4:** 

**PDF:** 
